# Equitable buyouts? Learning from state, county, and local floodplain management programs

**DOI:** 10.1007/s10584-022-03453-5

**Published:** 2022-10-26

**Authors:** Linda Shi, Anjali Fisher, Rebecca M. Brenner, Amelia Greiner-Safi, Christine Shepard, Jamie Vanucchi

**Affiliations:** 1 Department of City and Regional Planning, Cornell University, 213 West Sibley Hall, Ithaca, NY USA; 2 King County Government, Seattle, WA USA; 3 Jeb E. Brooks School of Public Policy, Cornell University, Ithaca, NY USA; 4 Department of Public and Ecosystem Health and Department of Communication, Cornell University, Ithaca, NY USA; 5 The Nature Conservancy, Gulf of Mexico Program, Key West, FL USA; 6 Department of Landscape Architecture, Cornell University, Ithaca, NY USA

**Keywords:** Flood, Social vulnerability, Climate adaptation, Buyouts, Managed retreat, Relocation

## Abstract

Climate change-exacerbated flooding has renewed interest in property buyouts as a pillar of managed retreat from coastal zones and floodplains in the United States. However, federal buyout programs are widely critiqued for being inaccessible and inequitable. To learn whether and how subnational buyout programs overcome these limitations, we examined five leading US state, county, and local buyout programs to see what they teach us about redesigning future federal policies. Our mixed-methods research used interviews and document analysis to develop case studies, juxtaposed subnational strategies against a review of critiques of federal buyouts, and focus group discussions with subnational buyout managers and experts to identify limitations of their programs. We find that subnational programs can be more inclusive and better respond to resident needs as compared to existing federal programs due to their access to dedicated, non-federal funding and their standing institutional status, which allows them to learn and evolve over time. Nevertheless, these programs lack coordination with and control over agencies that permit development and produce affordable housing. This gives buyout programs limited power in shaping the overall equity of who lives in floodplains and who has access to affordable, resilient housing after a buyout. Their experiences suggest federal programs can support managed retreat nationwide by increasing support for institutional and staff capacity at state and county levels, encouraging efforts to bridge institutional silos at subnational levels, and holistically mainstream climate considerations into regional floodplain development, affordable housing production, and flood risk mitigation.

## Introduction

Climate-exacerbated flooding has renewed interest in home buyouts as a pillar of flood risk reduction and managed retreat from coastal zones and floodplains in the United States. However, floodplain buyout programs, especially the country’s largest one funded by the Federal Emergency Management Agency (FEMA), have drawn widespread criticism for being overly bureaucratic and socio-economically and racially inequitable (Hino et al. 2017; Howell and Elliott 2018; Mach et al. 2019; Peterson et al. 2020; Elliott et al. 2021). A growing body of research examines how to reform federal policies, what policies to replace them with, and what barriers stymie policy implementation (Kraan et al. 2021; Mach and Siders 2021; Hino and Nance 2021). More research on past and existing buyout programs is needed to support policy learning and coordination (Greer and Brokopp Binder 2017).

In this paper, we examine five dedicated subnational (state, county, and local) buyout programs in New Jersey, Washington State, Charlotte-Mecklenburg County (North Carolina), Harris County (Texas), and Austin (Texas) to understand whether regional buyout programs offer alternative approaches that can inform either future federal policy reform or an expansion of subnational buyout and floodplain management programs. In addition to traditional federal funding support, which imposes rigid requirements, subnational buyout programs also draw on more flexible state or local funding. In studying these well-resourced programs, we aim to assess (1) whether and how programs with greater autonomy and resources have responded to issues of equity, justice, and resident needs, and (2) what the limits of their achievements suggest about what it takes to achieve equitable adaptation in the floodplain.

Our mixed-methods research draws on case studies of these buyout programs, interviews with managers and field staff, and focus group discussions with buyout managers, planners, and academics. Below, we first categorize critiques of how floodplain buyout programs inequitably affect flood-impacted households. We then briefly describe each program before juxtaposing their policies and strategies with the literature to determine the extent to which they overcome common critiques. Drawing on interviews and document analysis, we examine why it is that they can overcome some of the critiques (e.g., greater resident inclusiveness and responsiveness), but not others (e.g., access to infrastructure in the past and affordable replacement housing). We argue that these subnational programs are successful because they endure and therefore can learn and evolve. They also have dedicated funding that allows these programs to function flexibly and proactively. We conclude by highlighting the importance of federal and state support for institution building and bridging at subnational levels to transition towards revitalized floodplains and affordable housing in flood safe neighborhoods.

## FEMA buyouts and their inequitable effects

A buyout takes place when the government buys a property from its owner to reduce the risks they face living in a repetitively flooded site. Buyouts have been a strategy for flood risk reduction in the United States since the 1970s (Hino et al. 2017; Peterson et al. 2020). As climate change increases flooding damages, scholars and advocates argue the federal government should increase its funding for buyouts, which are less than 20% of FEMA’s overall flood risk reduction budget (FEMA 2022a, b; Hino & Nance 2021; Koslov 2016; Marino 2018; Siders 2019). Conceptually, buyouts are a triple win: vulnerable residents move out of harm’s way, the government reduces its liabilities, and land can be restored and increase the area’s resilience to future floods. Done well, buyout parcels can generate additional social and ecological benefits, from reducing urban heat islands to creating habitat corridors and public green space.

In this paper, we focus on FEMA’s Hazard Mitigation Grant Program (HMGP), which accounts for 70% of federally funded buyouts and has bought out over 43,000 properties since its inception in 1989 (Mach et al. 2019).^1^ FEMA funding is awarded to state or local governments, who must provide a 25% match, following a presidential disaster declaration (FEMA 2016). The program’s chief goal is to reduce the National Flood Insurance Program’s liabilities for helping insured homes rebuild after repetitive floods. Accordingly, homeowners are eligible for buyouts if they live within Special Flood Hazard Areas (FEMA 2015; HUD Exchange n.d.), their city or county participates in the National Flood Insurance Program (Maryland DEM n.d.), their home meets the cost-effectiveness requirement (usually, a benefit–cost ratio equal to or exceeding 1:1) (Maryland DEM n.d.), and they are a citizen of the United States (FEMA 2015). Following a disaster, state or local governments identify properties with willing sellers to acquire. Homeowners receive funding equal to their home’s pre-disaster fair-market value. The home is demolished, and land is permanently deed-restricted from development.

The program’s design produces interlinked implementation challenges. While highly bureaucratic, the process is not standardized, leaving much room for interpretation and bias. Since federal funding only becomes available after a disaster, states, counties, and cities typically create ad hoc, temporary administrative offices to manage buyout funding. Reactionary offices must navigate complex paperwork requirements (which are being reformed under the Biden Administration), determine which neighborhoods and homes to prioritize, help residents make difficult decisions, and manage the land after a buyout (Bullard 2018; Elliott et al. 2020). For instance, both New York City and New York State created new buyout program offices after Hurricane Sandy, which were separate from ongoing state and county FEMA buyout processes and ended seven years after the storm. Paul Lozito, Director of Housing Policy and Affordable Housing for the New York Governor’s Office of Storm Recovery, created after Hurricane Sandy, noted that “At every step, we had to create a new process. And on the ground, everything was new. The city had never had to remove an entire neighborhood before. There was no past precedent. And we had to do everything in short order” (Lozito 2022). Each time, programs must learn and establish bureaucratic functions, staff, and relationships, all of which affect their ability to coordinate, collaborate, learn, and expand services, especially to vulnerable, harder-to-reach groups. Moreover, while the federal government provides 75% of the buyout funding, local tax rolls may be permanently reduced when valuable real estate is removed from the city’s building stock (BenDor et al. 2020). Total project costs add a further 25% over property buyout costs, which local governments must shoulder (Curran-Groome et al. 2021). These fiscal impacts further complicate local government participation and neighborhood prioritization.

The resulting HMGP program has been heavily criticized for having disproportionately negative impacts on disadvantaged households (e.g., Kraan et al. 2021; Elliott et al. 2020). First, the HMGP often violates principles of distributive justice, which argue that the distribution of resources should be made such that they benefit, rather than worsen, the outcomes of the most disadvantaged (Rawls 1971; Bullard 2001). While not all residents want to relocate, some believe buyouts allow them to leave a place with traumatic memories, recoup financial investments, improve their quality of life, or protect themselves or others from another disaster (Koslov 2016). However, the HMGP either is legally restricted to or procedurally favors single-family homeowners, nuclear households with a single head of household, and those with a clear mortgage, ownership documentation,^2^ US citizenship, and the ability to engage in a lengthy, burdensome process (Bukvic and Borate 2021; Seong et al. 2022). Households with upside down mortgages where the debt exceeds pre-disaster market value (an increasingly common condition of homes devalued by flood risk or flood insurance) are ineligible since the buyout would not resolve the debt. Therefore, the most financially precarious residents — renters (who comprise 28% of floodplain residents and may be displaced by landlords to take a buyout), immigrants, households living in multigenerational or cooperative housing arrangements, Indigenous groups or informal residents with different property rights regimes, and those with upside down mortgages — receive the least support and may feel trapped in places with high flood risk (Dundon and Camp 2021; Pastor et al. 2006; Marino 2015; Rosoff and Yager 2017).

Paradoxically, federal buyout programs can also be a means by which to dismantle poor communities or clear neighborhoods perceived as blighted (Bullard and Wright 2012). FEMA’s cost benefit analysis targets homes costing less than $323,000 (FEMA 2022a, b). An analysis of FEMA’s 43,000 buyouts to date found that while wealthier counties implemented more buyouts, bought-out properties within those counties were relatively poorer, with lower education levels, lower English language proficiency, and greater racial diversity (Mach et al. 2019). This has a disproportionate effect whereby residents at the greatest disadvantage bear the heaviest burden in terms of buyouts and relocation (Shonkoff et al. 2011; Levy and Patz 2015; Burkett 2018; Supekar 2019). While a buyout could in theory support housing mobility, residents in hot housing markets increasingly have difficulty buying a comparable home nearby (Finley 2022; Freudenberg et al. 2016; Thompson 2018). Federal programs do not fund relocation assistance, financial counseling, or provide real estate services. Given race-based homeownership gaps in the United States (Lerner 2020), buyouts can disrupt Black and Hispanic groups’ hard-won housing equity and generational wealth-building if they are not able to afford a comparable home in hot housing markets. By contrast, residents of wealthier, whiter communities are more often seen as too valuable to be relocated, and instead are offered seawalls, funding to elevate homes, or drainage infrastructure (Siders and Keenan 2020; Nance et al. 2022).

Second, lack of transparency in buyout decision-making raises fears of procedural injustice, whereby the process of decision-making disenfranchises those who historically have had the least voice (Young 2002; Greer and Brokopp Binder 2017). Households typically do not understand how home values are appraised (de Vries and Fraser 2012), who gets a buyout, and why, especially since decisions reflect factors like the existence of protective infrastructure, cost of future protection, ability to reduce expenses on public services, ecosystems benefits, historic preservation value, and political or community opposition. Residents who lack financial flexibility feel their participation was involuntary and as such lack agency in their lives and relocation (de Vries and Fraser 2012; Baker et al. 2018). Opaque processes and time compression can “create the appearance of unfairness” and in turn can undermine “faith in the system” and lead to lower participation rates and half-vacant neighborhoods (Siders 2019, p. 248). For those participating, 5 years is the median time for a federal buyout to be processed (Weber and Moore 2019). This places an extraordinary burden on residents who lack savings or access to alternative housing. Many instead elect to rebuild or sell their home before a buyout happens. Poorer households wait longer to receive less money and end up worse off, raising fears that wealthier households (as well as wealthier local governments) are better able to navigate the complex and biased systems (Mitsova et al. 2019; Siders 2019). Indeed, low- to moderate-income households are less likely to receive full and timely compensation because they are less likely to afford or successfully mount an appraisal appeal (Muñoz and Tate 2016).

Third, buyout programs do not account for more holistic dimensions of flood vulnerability, from the historic drivers of why households live in flood prone areas without sufficient protective infrastructure, to questions of household well-being after relocation, to concerns of gentrification or displacement effects of relocation. People’s vulnerability to floods and flood risk mitigation efforts reflect not only the elevation of their home but historic drivers of social, economic, and spatial inequality (Bullard and Wright 2009; Smiley 2020; Tate et al. 2021). Housing discrimination studies in the southeastern United States show racial segregation by altitude, with Black communities at lower altitudes where they are more at risk to floods and lower property values (Ueland and Warf 2006). While most buyouts take place outside of formerly redlined areas, those within redlined zones disproportionately take place in zones redlined as “C” and “D,” primarily inhabited by low-income and racialized minorities (Zavar and Fischer, 2021).

Given the shortage of safe affordable housing, buyout programs could potentially shift vulnerable households to other places of risk. As a program focused on property rather than people, FEMA does not require people move to flood-safe neighborhoods nor does it track buyout households’ relocation details. One investigation of relocation from Staten Island after Hurricane Sandy found that 99% of 323 households studied relocated to areas that scored worse in one of multiple metrics used to measure social vulnerability and 20% to places with greater flood risk (McGhee et al. 2020). In Houston, after Hurricane Harvey, comparatively wealthier buyout participants were more likely to relocate closer to their original communities and thereby retain community ties. By contrast, lower-income households became more dispersed, moving further away from their original neighborhoods in search of affordable housing, thereby losing place and community ties (Elliott et al. 2021). Loss of community ties and moving to less stable locations can have both mental and physical health implications (Binder et al. 2019; Umberson and Montez 2010; Hurd et al. 2013).

Buyouts reduce the risk of property loss and human suffering in flood-prone areas and may be one of the most effective tools available to reduce risk and provide resources for disadvantaged communities (Freudenberg et al. 2016). However, the inequities built into the design of the FEMA program result in distributive and procedural inequities that compound rather than redress long-standing structural drivers of inequity. These trends suggest that even if more funding were allocated for buyouts, they would not necessarily produce a socially equitable outcome. How can the current program address critical limitations in distributive and procedural injustices? How have long-standing, reputable programs responded to equity concerns? And what do their experiences teach us about the possibilities and limitations of more equitable flood risk mitigation moving forward?

## Methods

We respond to these questions by studying five state, county, and local buyout programs, examining how they respond to the social equity concerns raised by FEMA-funded buyouts, and exploring opportunities to redress remaining gaps. Two are state-level programs (New Jersey’s Blue Acres and Washington’s Floodplains by Design), two are county-level programs under Harris County Flood Control District and Charlotte-Mecklenburg County Storm Water Services, and one is a city program in Austin, Texas. Together, they comprise the five most prominent US programs of their kind by size, with standing operations (as opposed to being triggered by a federally declared disaster), and a reputation for innovation (Spidalieri et al. 2020; Peterson et al. 2020). Each has developed their own in-house program to gain agency over the federal buyouts and flood hazard mitigation process. Each has a dedicated source of funding (including state allocations, utility or property tax surcharges, and bonds), though most continue to rely on a mix of federal, state, and local dollars (see Peterson et al. 2020 for review of local sources of buyout funding nationwide). We posited that these programs’ higher staff capacity, opportunities to experiment and institutionalize learning, and partial flexibility from federal funding constraints allow them to respond more effectively to local needs and priorities, including for social well-being and equity.

To understand the opportunities and constraints these programs face, we conducted case studies on the history of their formation, structure, operational practices, policies, achievements, and evolution, drawing on documents, web searches, and interviews. We interviewed 25 directors, managers, real estate experts, and case workers associated with buyout programs, as well as staff from The Nature Conservancy (TNC) between 2020 and 2021.^3^ We then used comparative matrices to analyze the extent to which programs responded to social equity concerns identified in the literature. For most cases, we interviewed staff a second time to probe how programs address eligibility, resident engagement, post-buyout relocation outcomes, resident concerns, the impact of national reckoning on race and equity, and surprises in their work. This analysis revealed that while many programs had developed discrete responses to specific equity considerations, all had difficulty responding to broader relational dimensions of justice that fell outside the narrow purview of buyout programs and the organizations where they sit.

Finally, in 2021, we conducted three focus group discussions with a total of 13 buyout managers, planners, and academics from Charlotte-Mecklenburg, Houston, and Washington to build a collaborative perspective. These conversations asked participants to reflect on achievements and barriers, and suggest directions for policy and practice to include inequitable flood risk and relocation vulnerability. Focus group facilitators asked participants what issues they struggled with in addressing floodplain risk, what barriers they encountered to addressing equity, what relationship exists between affordable housing and flood risk in their region, and whose responsibility it is to stop the cycle of housing vulnerability and promote safe, equitable neighborhoods. Each focus group included three to six participants, was limited to two hours, and was recorded, transcribed, and coded using MAXQDA software. Although most interviewees gave permission to be quoted, we have only referenced the name of their role. All interviews and focus groups took place over video conferencing due to COVID-19-related travel restrictions.

This project was limited to speaking with buyout managers and staff and a few academics and staff of nonprofits. It was beyond our scope to interview or survey a sample of buyout participants, whose contact information most of these programs do not track after relocation. This may result in an incomplete picture of the equity impacts of the buyout implementation process. To overcome this limitation, we critically assess these programs against the literature and other interviewees’ reflections.

## Snapshots of leading subnational buyout programs

### Austin Watershed Protection Department (WPD), Texas

Austin experiences extreme fluctuations in rainfall. With floodplains covering 10% of its land, the city is vulnerable to drought and flash floods, which climate change will exacerbate (Hayhoe 2014). Austin’s Watershed Protection Department formed in 1991 in response to growing concern about the impact of development on erosion and local water quality. It aims to restore natural floodplain functions to protect people and property using a suite of flood risk mitigation tools. While WPD had long managed creeks and drainage infrastructure, Hurricane Harvey led WPD to make buyouts a core strategy. To achieve Austin’s Watershed Protection Master Plan, WPD has bought out over 800 properties, identified based on an assessment of flood risks and potential solutions. Most are funded by a parcel-level drainage (stormwater) fee levied on impervious coverage, supplemented by municipal general obligation bonds, voter-approved bonds, and grants from FEMA’s HMGP and the United States Army Corps of Engineers (USACE). WPD is recognized for modeling its relocation benefits after the federal Uniform Relocation Assistance and Real Property Acquisition Act (URA) of 1970, which provides real estate and relocation assistance to people displaced by eminent domain (FEMA 2015). FEMA’s program requires voluntary participation in relocation, rendering participants ineligible for the URA. However, WPD applies URA’s formula and draws on its own funding sources to help owners bridge the cost between their old and new home. The program is not guided by explicit social equity goals or social vulnerability metrics (Buyout Manager Interview 2020).

### Harris County Flood Control District (HCFCD), Texas

Harris County’s flat prairie lowlands are crisscrossed by bayous and marshes. Hurricanes and extreme rainfall regularly strike. Houston, which is nearly coterminous with Harris County, has experienced 40 federal disaster declarations since 1953, including seven for floods between 2016 and 2021 (FEMA n.d.), the largest of which (Hurricane Harvey in 2017) flooded a third of Houston and caused $125 billion in damages (Blake and Zelinsky 2018). In 1937, Texas created the HCFCD to help USACE build dams, reservoirs, seawalls, and other flood infrastructure. Its role expanded to floodplain management and voluntary buyouts in the 1970s–1980s as urban sprawl increased flood exposure. Local bonds, FEMA, HUD, and USACE support HCFCD and Harris County voters approved $2.5 billion in bonds in 2018 for HCFCD to deploy flood control projects.

As America’s largest buyout program, HCFCD had spent $340 million buying out over 3,000 properties before 2018 and plans over 3000 more under the new bond (Elliott et al. 2021). After community groups claimed that infrastructure investments were disproportionately deployed in wealthy white areas, the County Commission asked HCFCD to account for social equity in its project prioritization scheme (Flavelle 2020; Nance et al. 2022). HCFCD’s policy documents set the goal to “prevent the worst impacts on people first (‘worst first’ approach)” (HCFCD 2019). In other words, the people likely to experience the worst impacts are attended to first. It identifies priority areas for flood mitigation projects using the Center for Disease Control’s Social Vulnerability Index, depth within the floodplain, existing levels of drainage service, project efficiency, partnership funding, potential for multiple benefits, and long-term maintenance costs (HCFCD 2019).

### Charlotte-Mecklenburg County Storm Water Services (CMSS), North-Carolina

Mecklenburg County sits in the Catawba River Basin, which has a network of urban creeks and lakes that flash flood regularly during hurricane season. Climate change has increased heavy downpours in Charlotte, the county’ largest city, by 86% since 1950 (Climate Central 2015). CMSS is a joint county-municipal stormwater utility whose mission is to manage runoff and reduce flood risk, including through voluntary home buyouts. It was created in 1999 in response to two devastating floods that caused over $60 million in property damage and flooded homes outside the established floodplain. This prompted 30 to 40 exasperated residents to show up to a county commissioner’s meeting with mops and buckets, demanding better stormwater protection (del Charco 2018).

After relying on FEMA’s HMGP funding and floodplain maps for a decade, CMSS grew frustrated with its limitations. Local floods were not big enough to trigger federal disaster declarations, most flooded properties did not meet FEMA buyout eligibility criteria, cost-benefit analysis did not account for community-level benefits, and buyout times were so long that most residents opted to rebuild. Realizing that it needed to work more swiftly and think locally, CMSS shifted their approach from flood prevention to damage mitigation (Buyout Manager Interview 2020). It established a stormwater fee that generates most of its $4 million annual budget for buyouts that do not meet FEMA criteria (Buyout Manager Interview 2020; City of Charlotte n.d.). To date, it has bought out over 400 properties (City of Charlotte n.d.) and is recognized as a model for adopting a data-driven approach that enjoys a high degree of public approval. However, it has not adopted explicit social equity goals or social vulnerability metrics.

### New Jersey Blue Acres

New Jersey, the densest state in the country, is also one of the most flood-prone, with heavily developed floodplains near New York City and development along the sandy coastal plain (CDC 2011; U.S. Census Bureau 2021). The combination of increased rainfall and development in the region have contributed to eight federal flood-related disasters in New Jersey since 1962 (FEMA n.d.). Hurricanes and tropical storms are expected to reach New Jersey’s latitude more regularly under climate change, causing heavier rainfall, storm surge, and riverine floods (NJ DEP 2020). In 1961, the state Department of Environmental Protection founded the Green Acres program to acquire and preserve undeveloped land to create a statewide open space network. In 1995, it established Blue Acres as an extension and partner of Green Acres to address flood prone properties. Blue Acres acquires contiguous parcels to maximize ecological benefits and uses voluntary buyouts to return properties to nature and restore them to passive recreational green space.

Established through three bond acts totaling $36 million, Blue Acres was initially entirely state-funded before securing $273 million from FEMA and HUD after Hurricane Sandy in 2012. As of 2020, 6% of New Jersey’s corporate business tax supports the program, which has enabled Blue Acres to do buyouts on a more strategic, longer-term basis (Spidalieri et al. 2020). These funds enable the program to hire a diversified staff of legal, real estate, financial, and policy experts who help homeowners overcome hurdles and relocate faster than through the federal process (Weber and Moore 2019). Blue Acres’ director, Fawn McGee, has led the program since 1995, developing over time a team of “dedicated and passionate staff” who are in “constant communication” with FEMA and New Jersey Office of Emergency Management (FEMA 2021a, b). In total, Blue Acres has secured funding for 1200 properties and demolished 700 properties (ULI 2020), mostly in inland municipalities. It has not adopted explicit social equity goals or social vulnerability metrics.

### Washington Floodplains by Design

Flooding is the State of Washington’s most frequent and expensive natural hazard, with 30 federal flood disaster declarations since 1956 (FEMA n.d.), affecting every county at least once (TNC n.d.). Climate change exacerbates these floods, as previously snow-based systems become rain-based ones, thereby increasing flood exposure. Advocacy by environmental groups and First Nations led the state to create the Department of Ecology (DOE) in 1970 to administer and enforce state floodplain and environmental laws, with a focus on restoring salmon health (DOE Interview 2020).

In 2013, The Nature Conservancy, DOE, and Puget Sound Partnership created Floodplains by Design, a floodplain restoration program that shifted from uncoordinated “postage stamp” restoration projects to wide-reaching, community-led restoration projects with enough scale to make a difference (DOE Interview 2020). With $45 million a year in state funds, the program promotes the concept of integrated floodplain management, salmon recovery, and habitat restoration. It provides competitive grants for local governments and First Nations to implement large-scale restoration and flood mitigation projects (DOE Interview 2020). Buyouts are a small component of the program, which encourages and enables communities to solve intersecting water, flood, fish, and farm challenges. It has focused on building local capacity and partnerships — between agencies, jurisdictions, disciplinary silos, and stake or rightsholders — so that integrated floodplain management principles can take root locally.

As of 2021, Floodplains by Design has reduced flood hazards in 59 communities, reconnected more than 7000 acres of floodplains to their river systems, restored 50 miles of river habitat, and removed over 2000 homes and structures from high-risk flood zones (Floodplains by Design 2021). Most of its work is in rural areas, where floodplain management and land conversion are cheaper and easier. It has done little work in urban areas, nor does it focus explicitly on equity.

## Enhancing programs’ fairness

While only Houston’s program has adopted formal equity objectives in their mission statement, all five programs have developed strategies to address inequitable dimensions of buyout programs. These strategies try to improve distributive equity by diversifying criteria to include more residents and business owners, and procedural equity by enhancing public communication and engagement. However, programs’ approaches to equity narrowly define the buyout process and struggle to acknowledge or address factors causing inequitable exposure to flood risks or household vulnerability after relocation.

### Extending distributive equity

Programs in Austin and New Jersey have diversified buyout eligibility by expanding beyond FEMA’s typical support for landlords and renters and letting those with upside down mortgages participate. For instance, Austin’s program pays for relocation advising, a new home (including the difference between the original and a comparable replacement), property appraisal, moving, and closing costs, and up to $25,000 to reestablish businesses. It also helps renters participate in buyouts by offering benefits through the federal URA standard (Buyouts Manager Interview 2020; Buyouts Real Estate Officer personal communication 2022). Similarly, New Jersey’s program has staff who specialize in tenant relocation and work directly with landlords when buying out a rental. In-house real estate and financial experts work with public and private financial institutions to secure debt forgiveness for upside down mortgages, without which 15% of participants would be unable to accept the buyout (Spidalieri et al. 2020).

Programs have also diversified criteria for cost-benefit analysis to better assess when a buyout is advisable. As Austin’s manager noted, we “realiz[ed] that … just looking at cost is not always the best indicator” (Buyouts Manager Interview 2020). Instead, Austin uses a matrix when deciding where to do a buyout — including cost effectiveness, permitting feasibility, and potential for habitat restoration and public open space. The final decision reflects an outreach process about which option will best suit a particular household. Similarly, Charlotte-Mecklenburg uses a holistic scoring system that captures local risks, land use priorities, and vulnerabilities. They identify potential mitigation strategies based on the cost-effectiveness of a buyout and probability of future flood risk and damages. Then they prioritize properties for assistance given their value and benefit to the community (such as civic centers, historic sites, or proposed recreation trails). Its program director notes that broadening evaluation criteria to include an array of locally relevant metrics besides property value is one way that equity is integrated into the system (Buyouts Manager Interview 2020). Nevertheless, metrics like these in Charlotte-Mecklenburg and Washington State can end up prioritizing places requiring fewer easements or buyouts. In some cases, this meant, “We were not equitably serving the community, we were just making it easier on ourselves” (Program Director Interview 2020).

Some programs also use discretionary funds to diversify alternatives to buyouts that help residents stay in their homes longer or age out of their homes. Charlotte-Mecklenburg’s retroFIT program offers eligible homeowners local grants to cover 75–95% of expenses to flood-proof or elevate their home. Funded mostly by the county’s stormwater tax, it aims to protect floodplain property owners who are ineligible for a buyout or other mitigation efforts. Occasionally, Charlotte-Mecklenburg and Austin use leasebacks (where the government purchases the home and residents lease it) to give households, especially people over 65, time to move.

### Enhancing procedural equity

These programs conduct extensive public engagement by establishing clear guidelines, criteria, and application processes that enable participation and offer transparent support. Personal advocates help reduce wait times and build trust in the process, which increases participation in buyouts (De Vries and Fraser 2012; Binder and Greer 2016). Austin and New Jersey assign residents a real estate consultant or town liaison, respectively, to help navigate the buyout process (Real Estate Services Officer Interview 2021). Charlotte-Mecklenburg’s peer-to-peer program has past buyout participants share their experience with eligible households. In a nod to “participatory budgeting,” CMSS gave communities monopoly money and asked where they would invest and why (Program Director Interview 2020). Based on this input, projects are now weighed by whether they connect a greenway, expand public green space, or preserve a historic or culturally significant structure. Residents who feel that buyouts create community benefits are more likely to perceive it as legitimate and participate in the process (Program Manager Interview 2020). Washington State’s program works with The Nature Conservancy to publish success stories and a web map of all project locations, scopes, and funding. In addition to a 125-page Flood Risk Assessment and Reduction Community Guidebook (CMSS 2020) that details the entire process, Charlotte-Mecklenburg’s website also offers an interactive flood risk mapping tool so people can check their risk level and share successful projects.

All five programs proactively buy out properties based on established plans rather than solely reactive, post-disaster efforts. This proactive approach shortens wait times, reduces emotional and financial stress, and raises awareness to help communities prepare emotionally and financially for a buyout. This planning model also helps build trust and enables residents to exercise their agency, participate in the planning process, and make an informed decision about their future. Since federal disaster funding has historically only become available post-disaster, local funding sources are key to proactive efforts.

### Challenges acknowledging pre-disaster injustice

While buyout programs have enhanced their inclusiveness and responsiveness, they did not expressly acknowledge in the interviews or published materials the historic contributors of uneven flood risk vulnerability. For instance, Houston’s “worst first” policy is the most explicit of these five programs in prioritizing social equity, but still focuses solely on present-day risk assessment. This present-day focus neglects racial, political, economic, and infrastructural legacies that shape people’s lived experiences. The lack of recognition of historic decisions, including for housing and flood control, affects project prioritization projects and community trust building. If there is a goal of reparative justice, accomplishing that becomes much more difficult if there is not a shared sense of what needs repairing.

These silences differ from the challenges that academic, journalist, and other observers identified. For instance, an academic focus group participant conducted a study in Houston showing that two communities that flooded to similar depths during Hurricane Harvey received very different post-disaster recovery packages, split along race and class lines (Nance et al. 2022). Homes in Brays Bayou — a white and Latinx middle/upper-income neighborhood that had received flood protective infrastructure for decades — were much more likely to receive home elevation grants because they were seen as having greater long-term resilience. Only 72 were bought out. By contrast, homes in Greens Bayou — a predominantly Black and Latinx working class neighborhood — were seen as “hopelessly deep in the floodplain.” Here, households were much more likely to be offered a buyout, and 1,154 homes in Greens were demolished (Nance et al. 2022). While many factors may have contributed to these different packages, such disparities are likely to shape communities’ perceptions of why they were flooded and what options they were offered. Efforts to promote reparative justice (Williams 2020) and attend to historic trauma (Poe 2022) can deepen buyout programs’ fairness. However, the language and ideas for such engagement appear to be challenging for agencies to incorporate.

### Struggling with safe, affordable relocation housing

Programs also have struggled to link buyouts with post-relocation housing that is affordable and safe, given that such housing is in short supply (Jacobson 2021; Worland 2021). While buyouts are gaining support, they can exacerbate local housing shortages. An Austin WPD real estate expert noted, “We are not helping the situation. Yes, we’re making people safer, and taking out dangerous housing stock, but in doing so we’re driving prices up…it’s a net decrease in the housing stock” (Interview 2021). Austin is unique in trying to help households relocate within the city by funding the price difference between their current and future homes. Even so, interviewees questioned the long-term feasibility of this strategy given how expensive housing has become in Austin. There is a clear need to complement buyouts with building affordable and resilient housing in cities or regions that have significant flood risks. However, such efforts are just emerging,^4^ and are often not linked to the buyout programs we studied.

Programs also have not been able to help buyout recipients relocate close to each other or to their original community to sustain social ties, community identity, and place attachment. Like FEMA, each program conducts individual property buyouts rather than blocks or neighborhoods. None of the programs address the psychological, health, symbolic, or emotional aspects of “root shock” (Fullilove 2016), for instance, through commemoration, resources for healing, or sustaining or building community in relocated sites (Agyeman et al. 2009). Nor do they support place detachment from communities or the stress of rebuilding networks. Coordination with various entities that address components of displacement could be a path forward.

## Institutional enablers, gaps, and barriers

### Institutional enablers

Two critical factors have enabled these programs to develop equity-oriented strategies. First, whereas most buyout programs emerge after a disaster, these programs’ continuous operations have helped them monitor, assess, and learn from past experiences. As standing programs, they are able to develop long-term plans and a level of operational capacity to respond to equity, affordability, and resident well-being. In interviews, buyout managers consistently spoke of how they learned on the job, how their programs’ benefits and eligibility criteria evolved as they had time to consider additional priorities (versus the rush of a post-disaster scenario), and how their own self assessments and reflections contributed to course adjustments. In Austin, Charlotte-Mecklenburg, and New Jersey, program staff highlighted how important it was to develop an ecosystem of relationships with other government agencies and develop a trustworthy reputation among residents. Agencies’ sustained presence also enabled watchdog and advocacy groups to assess them and push for institutional change. In Houston, for example, advocacy groups assessed HCFCD’s inequitable practices and pushed for change. In Washington State, The Nature Conservancy played a partnership role in advocating integrated watershed restoration.

Second, each of these programs has dedicated non-federal funding that (1) covers FEMA’s requirement for local cost-share, and (2) enables them to go beyond FEMA criteria in eligibility and forms of support. The options for generating resources vary. Charlotte and Austin raise funds through a stormwater utility tax on impervious surface, Harris County issued $2.5 billion in green municipal bonds, New Jersey uses a state corporate business tax, and Washington relies on state funding. These funds support locally identified priorities, some of which are common across the sites: housing affordability and support for renters, landlords, and upside-down mortgage holders. Others are more specific: prioritizing “worst first” neighborhoods in Houston, enabling greenway expansion in Charlotte-Mecklenburg, and supporting salmon protection in Washington. State funds are especially helpful for small municipalities that lack the necessary resources or capacity to develop long-term, multi-benefit buyout strategies. They can also reduce paperwork and fund such projects with one funding application. For example, Floodplains by Design enables local governments to fund salmon recovery, riparian restoration, levee setbacks, protecting river-adjacent neighborhoods, conservation easements, and buyouts (Program Manager Interview 2020).

### Institutional and systemic gaps

Even with dedicated and often innovative funding and a stable presence, these programs face challenges with incorporating equity considerations, often related to a lack of familiarity with the concepts, difficulties communicating and engaging with residents, and having inadequate training. Many interviewees and focus group participants noted that conversations with residents about buyouts, equity, and social vulnerability are challenging. Groups these programs found especially hard to engage included immigrants in Texas, farmers in Washington, and Tribal governments. Such engagement, interviewees noted, was difficult because they, as engineers and natural scientists, were never trained to deal with difficult social, emotional, and racial issues in their professional practice.

For instance, in Charlotte-Mecklenburg, interviewees realized their “neighborhood change score,” ostensibly created to avoid buying out neighborhoods that were more likely to “flip” and gentrify, could actually lead to exclusionary policies. “We can make numbers do anything. But is the change score what we should be using, or is it something else? And isn’t it better to have the conversation about should there be something else with people who know more about racial inequity and inclusion? We don’t have that expertise” (Program Director Interview 2020). This led CMSS to hire a facilitator to address issues of distributive injustices and invest in working with communities to consider what metrics would be appropriate to avoid further marginalizing disadvantaged groups.

Interviewees were often cognizant that they were likely not alone in dealing with these challenges. They expressed an interest in learning and growth, including an increasing openness to attending to the unintended consequences of various practices. Some were eager to participate in our research to learn more about how others were dealing with these questions. As Austin’s buyout manager observed, “A lot of people are looking to us to learn, and that’s left us scrambling for who to talk to [for us to learn from]. I think we need to branch out beyond floodplain managers. Our culture of buyouts, it’s tied into affordable housing, into racial equity that goes beyond flood risk” (Interview 2020).

### Organizational barriers

Finally, in focus group discussions, participants underscored the need for interdisciplinary and intergovernmental collaboration across levels of government and across local government agencies to overcome the narrowness of buyout programs’ purviews. Regardless of whether agencies endorsed equity statements or adopted equity-promoting policies, these broader contexts and policies can have even greater influence on the equity and justice of who is exposed to flood risk and who has access to flood protection and safe housing. Many participants noted that managed retreat needs a multidisciplinary approach beyond what is currently in practice. One participant observed (2021), “The problems didn’t start with the storm. They don’t end with the recovery money.… It’s beyond what a flood control district can really do.”

Yet, despite seeing the need for coordination, buyout programs struggled to change housing or floodplain development. For instance, planners and developers in Washington State continue to encourage development in areas that local governments had identified as high-risk. Buyouts staff in Austin and Charlotte-Mecklenburg were especially concerned with housing affordability for relocated residents and gentrification impacts on neighborhoods near green investments. Others noted the need to involve agencies that better understand community dynamics to facilitate effective relocation and rehousing efforts, and to assist with community outreach and education pre-buyout. In Charlotte-Mecklenburg, CMSS had formed a coalition with watershed, recreation, stewardship, and flood monitoring groups, not housing and community development. Indeed, in organizing focus group discussions, we could not find participants in these regions who worked on housing and felt buyouts were relevant enough for them to participate.

## Path forward

President Biden’s Administration has increased disaster funding, reduced their bureaucracy, and prioritized support for disadvantaged groups. Support includes $5 billion for FEMA, doubling the Building Resilient Infrastructure Communities (BRIC) program, requiring 40% of investments to benefit disadvantaged communities, and increasing federal cost share to 90–100% for buildings in socially vulnerable communities under the new Swift Current program (White House 2021; FEMA 2022b). These address many distributive and procedural justice concerns in the literature. However, they do not resolve constraints inhibiting subnational buyout programs from achieving greater equity outcomes, nor do they extend what made subnational programs effective to other geographies. Below, we identify additional ways for federal programs to make buyouts more equitable.

### Increase federal funding for institution building at state and county levels

Subnational programs’ long-standing operations helped them learn, evolve, and grow the staff capacity to do complicated, coordinated, and socially sensitive work. County and state-level programs also reduced inter-municipal disparities by providing specialized expertise in a one-stop shop. As flood risks intensify and expand geographically, more and more communities with limited capacity will need to contemplate and implement buyouts. While FEMA’s funding has increased, just 4% of BRIC funds disbursed in 2021 funded capacity building projects, while 87% went to mitigation projects (FEMA 2021a, b). More federal funding and technical assistance are needed to help state, county, or regional governments establish permanent offices that help local governments handle buyouts (Kraan et al. 2021; Peterson et al. 2020; Enriquez 2021).

### Create multi-sectoral programs at federal, state, and local levels to enable more integrated problem solving

Institutional silos at all levels of government prevent localities from planning for integrated development permitting, flood control, housing, and buyouts. For instance, HUD mentions FEMA just once in its 41-page national Climate Action Plan, even though climate change significantly affects existing and future affordable housing stock. At the local level, agencies managing buyouts have no control over floodplain development or relocation housing availability. Subnational buyout programs’ struggles in changing systemic inequity in flood vulnerability suggest that making federal buyouts more accessible to disadvantaged groups is insufficient. Rather, agencies like FEMA and HUD can help bridge these divides by coordinating funding opportunities so that communities can apply for integrated grants. For instance, FEMA, HUD, and the Environmental Protection Agency (EPA) could develop a joint fund for planning and integrating floodplain risk reduction, housing relocation assistance, affordable housing production, and ecological restoration. Even if structurally reorganizing FEMA and HUD at the national level is unlikely, they can help fund new organizations at state and county levels that plan for and fund integrated floodplain land use, housing relocation, and restoration.

### Allow local governments to spend federal funds more flexibly, including for pre-disaster mitigation

Subnational programs are effective because they have dedicated funding that they can spend flexibly. While new BRIC and Swift Current requirements are more inclusive, the HMGP (which received 69% of the $5 billion special allocation in 2021) still permits only 4% of its funds to be used for pre-disaster mitigation (White House 2021). Learning from subnational programs, FEMA could adapt HMGP criteria to better support residents with upside-down mortgages, non-citizens, and residents of multifamily housing. Furthermore, it can set mission- or performance-based targets — such as promoting the proportion or density of people outside the floodplains and increasing the percentage of disadvantaged residents living on higher ground — or reward localities for meeting their own locally-determined goals.

### Grow a professional cadre trained in inclusive and anti-racist planning and practice

Grow professional cadre trained in inclusive and anti-racist planning and practice. Cultural, social, anti-racism, and emotional competency is an essential part of any professional training. Federal agencies, academic institutions, and professional networks already are helping build subnational staff capacity to grapple with racism and classism in inequitable flood risk, infrastructure access, and processes of relocation. Building the capacity of the current Climate Corps or President Biden’s proposed Civilian Climate Corps to be effective communicators and facilitators also can promote the necessary workforce skills. In addition, efforts to bridge agencies as described above can also bring together complementary skills, for instance, by connecting technical buyout offices with community-oriented agencies that better understand how to engage people on issues of race and class.

There is an urgent need to scale up existing buyout programs to help bridge the vast gap between the 43,000 houses bought out over the last three decades, and the 22 million people at risk of a 500-year flood in 2020 or the 13–25 million households affected by six feet of sea level rise (First Street Foundation 2020; Mach et al. 2019; Robinson et al. 2020). Drawing on the experiences of lauded subnational floodplain buyout programs, our research demonstrates that institutional capacity is central to programmatic success and equity. Learning from their experiences suggests that federal agencies must not only disburse more funds but also strengthen the national institutional architecture to undergird the growing scale and frequency of climate impacts. A combination of strengthening state and county government offices, supporting cross-agency integrated programs and funds, increasing local implementation flexibility, and developing a national cadre of climate communicators can help meet the climate gap.

## Footnotes

1 The second largest is the Department of Housing and Urban Development’s Community Development Block Grant-Disaster Recovery (HUD CDBG-DR), which focuses on buying out low- and moderate-income households, helps communities “prevent or eliminate slums or blight,” and permits redevelopment of bought out properties. Others include those funded by the US Department of Agriculture. In this study, we focus on FEMA buyouts given the aim of our broader research agenda on post-buyout floodplain restoration.

2 Beginning in 2021, FEMA’s policies allowed households to show multiple forms of documentation to prove home ownership.

3 New Jersey Blue Acres was the only program where staff declined interviews. Here, we interviewed TNC staff and attended multiple presentations given by Fawn McGee, the Blue Acres program director.

4 An exception is Harris County’s Project Recovery program (run by the County Community Services Department, not HCFCD), which has linked its plans to buy out 2000 homes impacted by Hurricane Harvey with the HUD-funded Single Family New Construction Program. The program builds new or retrofits existing affordable housing (for those whose incomes are less than 120% of area median income) in mixed-use neighborhoods in less risky areas that are close to transit, jobs, and public services. Project Recovery plans to offer $19,800 in additional incentives for families to relocate within Houston (Project Information Officer Interview 2021).
